# Supplementary material for: Alterations of redox and iron metabolism accompany the development of HIV latency
Source: EMBO J. 2020 Mar 11;39(9):e102209. doi: 10.15252/embj.2019102209 (PMC7196916; doi:10.15252/embj.2019102209)
Supplement: Supplementary file 1 — Appendix [file EMBJ-39-e102209-s001.pdf]

Appendix

Table of contents

1. **Appendix figures S1 - S3**

2. **Legends of Appendix figures**

Appendix Figure S1

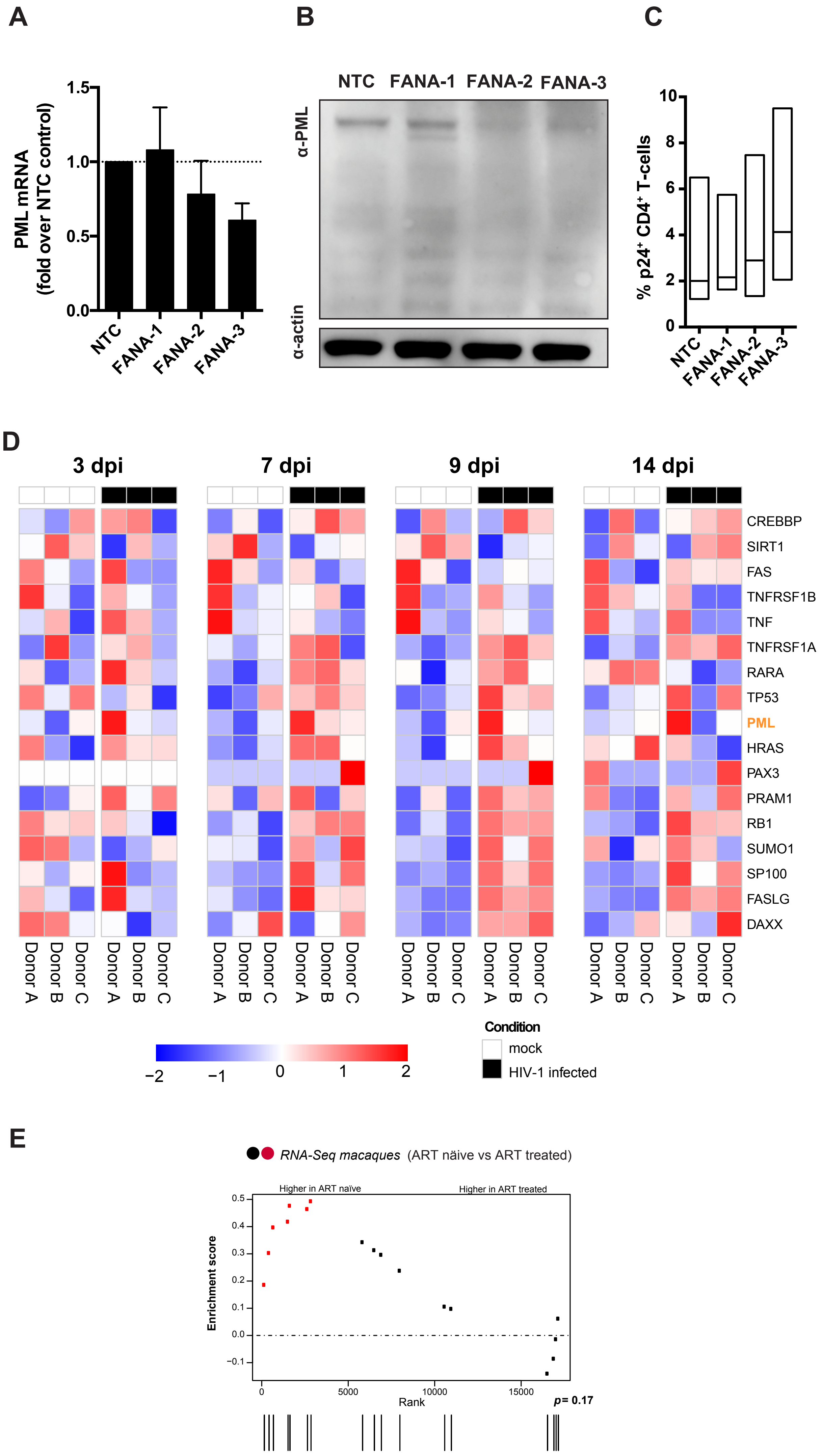

Appendix Figure S2

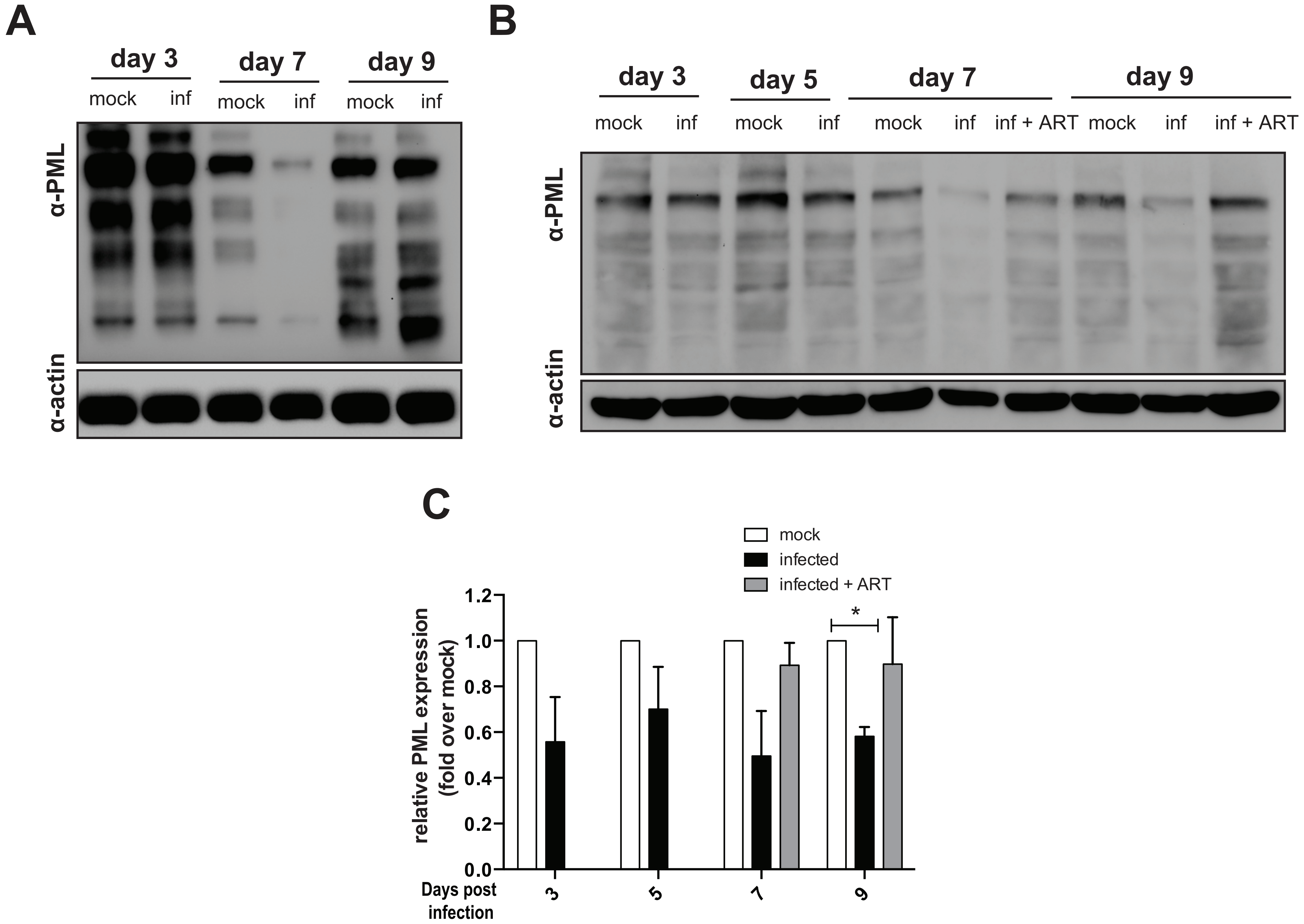

Appendix Figure S3

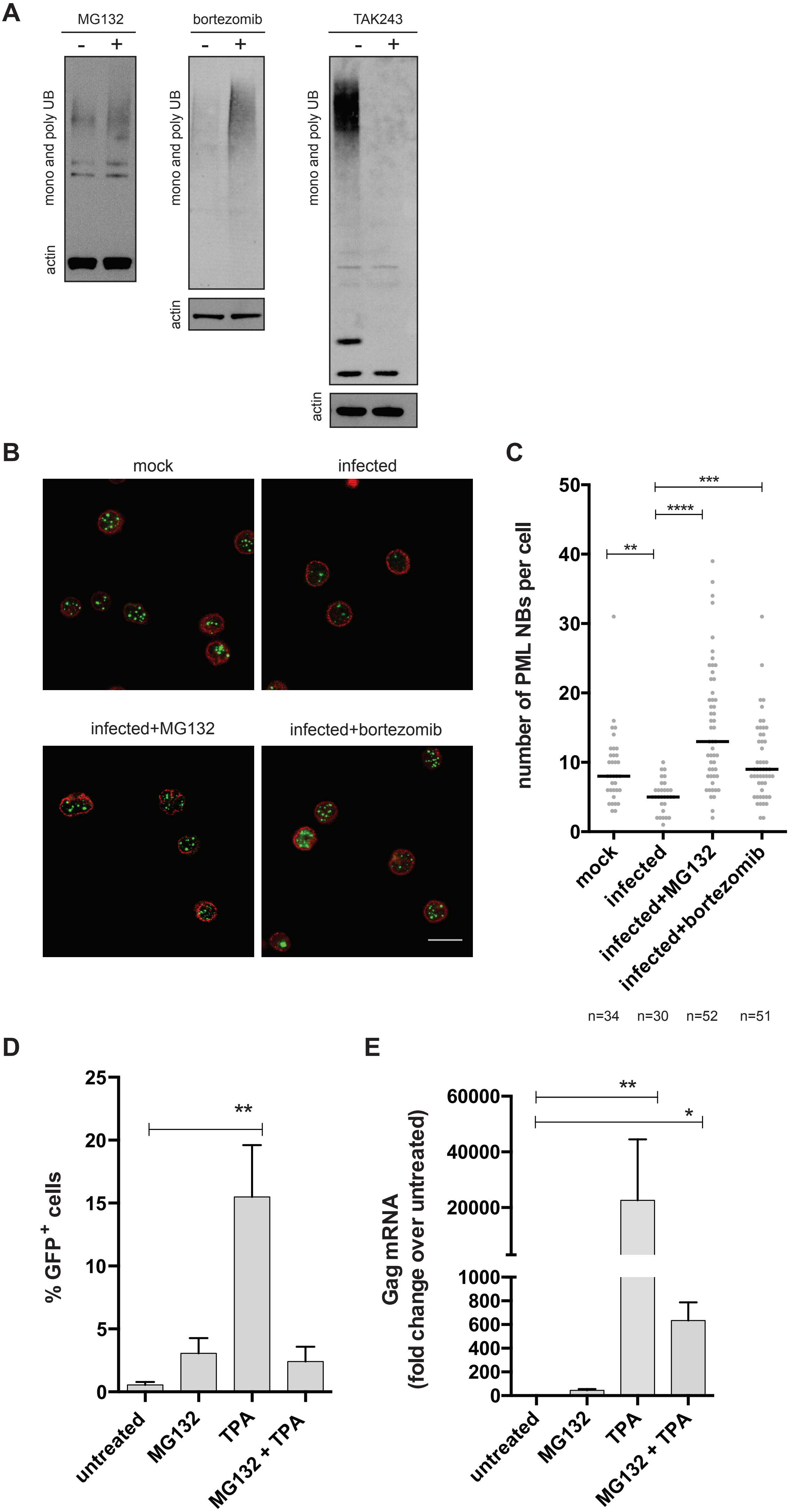

Appendix Figure S1. **Regulation of PML pathway and effect of PML knock down in the transition between productive and latent HIV-1 infection.** (A) Heatmaps of RNA-Seq data depicting the standardized expression of genes of the *Biocarta_PML_pathway* (17 genes, M4891) in HIV-1 infected and mock infected samples over time. Expression levels were standardized by calculating a Z-score [(mean gene expression - SD)/SD] for each gene in each time point. The PML gene is highlighted. (B-C) PML mRNA (B) and protein (C) expression in activated $CD4^{+}$T-cells treated for 72 hours with non targeting oligos or three different FANA oligos designed to silence PML. Data were measured by qPCR (B) and western blot (C). For (B), raw data were first normalized using 18S as housekeeping control and then expressed as fold mRNA expression in FANA PML vs non targeting oligo calculated as in (Livak & Schmittgen, 2001). Data are expressed as mean±SEM of three biological replicates (D) Frequency of $p24^{+}$ $CD4^{+}$ T-cells at 14dpi. Cells were treated at 11dpi with non targeting oligos or FANA oligos designed to silence PML. Data were measured by flow cytometry (mean±SEM of four biological donors).

Appendix Figure S2. **PML depletion is independent of cell viability and can be inhibited by ART.** (A) PML protein expression over time in mock infected and HIV-1 infected cells enriched for viability. Viability enrichment was performed as described in figure EV3C while protein expression was assayed by western blot. (B,C) Western blot (B) and relative expression (C) of PML protein over time in mock infected vs HIV-1 infected $CD4^{+}$T-cells with or without ART (added at 5 dpi). Western blot quantification was performed with Fiji-Image J (Schindelin et al. 2012), normalized over the housekeeping protein beta-actin and expressed as fold change over mock infected cells. Data are expressed as mean±SEM of 3 biological replicates and were analyzed by one-way ANOVA followed by Tukey´s post-test for multiple comparisons (only data 7dpi and 9dpi were compared). * $P<0.05$

Appendix Figure S3. PML degradation is mediated by the ubiquitin-proteasome axis. A) Western blot of mono- and polyubiquitinylated conjugates in latently HIV-1 infected J-lat 9.2 cells left untreated or treated for 4 hours to block proteasome function using MG132 (10µM) (left) or Bortezomib (100mM) (middle) or to block ubiquitination using TAK243 (10µM) (right). Representative immunofluorescence images (B) and quantifications (C) of PML NBs (green, maximal projection) and mab414/nuclear pore (red, one Z stack) in primary $CD4^{+}$ T-cells mock infected or HIV-1 infected for 7 days. Scale bar 10µm. D, E) GFP protein (D) or relative *gag* mRNA (E) expression in J-Lat 9.2 cells in which HIV-1 was latent or reactivated with TPA for 24 hours. Before TPA reactivation, cells were left untreated or pre-treated for 4 hours with MG132. GFP and *gag* expression were measured by flow cytometry and real-time PCR, respectively. Real-time PCR data were normalized using 18S as housekeeping gene. After normalization data were expressed fold mRNA expression over untreated as in (Livak & Schmittgen, 2001). Data were analyzed using Kruskal-Wallis (C)

or Friedman (D,E) tests. Black dash indicates median, n= number of PML NBs. (* P<0.05; ** P<0.01; ***P<0.001; ****P<0.0001).
