# Supplementary material for: Alterations of redox and iron metabolism accompany the development of HIV latency
Source: EMBO J. 2020 Mar 11;39(9):e102209. doi: 10.15252/embj.2019102209 (PMC7196916; doi:10.15252/embj.2019102209)
Supplement: Supplementary file 2 — Expanded View Figures PDF [file EMBJ-39-e102209-s002.pdf]

Expanded View Figures

Figure EV1. Primary CD4$^+$ T-cell model of productive and latent HIV-1 infection.

A Experimental design.
B Percentage of intracellular gag p24$^+$ CD4$^+$ T cells as measured by flow cytometry.
C Relative level of HIV-1 *gag* mRNA copies as measured by qPCR. Raw data were normalized as in Livak and Schmittgen (2001) using 18S or GAPDH as housekeeping control and then expressed as fold *gag* mRNA expression relative to values at 3 dpi.
D Gating strategy employed to sort primary p24$^+$ CD4$^+$ T cells: Cells were infected with HIV-1$_{NL4-3}$ and sorted between 5 and 9 dpi.
E Standard curve obtained by serially diluting DNA from sorted p24$^+$ cells with DNA from mock-infected cells. The $\log_{10}$ copy number of integrated HIV-1 DNA is plotted on the *x*-axis, while the delta CT value (calculated as in Tan *et al*, 2006) is plotted on the *y*-axis.
F Number of copies (Log$_{10}$) of integrated HIV-1 DNA per million CD4$^+$ T cells. Data were measured by Alu-HIV PCR and normalized using the standard curve obtained from sorted p24$^+$ cells.
G FISH detection of HIV-1 DNA at latency, i.e., 14 dpi (green: HIV-1; red: lamin; blue: DAPI). Scale bar = 2 μm.
H Effect of 48-h HIV-1 reactivation with α-CD3/CD28 beads or 10 μg/ml PHA at 14 dpi. Reactivation was measured by flow cytometry as percentage of p24$^+$ CD4$^+$ T cells.

Data information: Data in (B, C, and F) are expressed as mean ± SEM of 3 biological replicates.

© 2020 The Authors

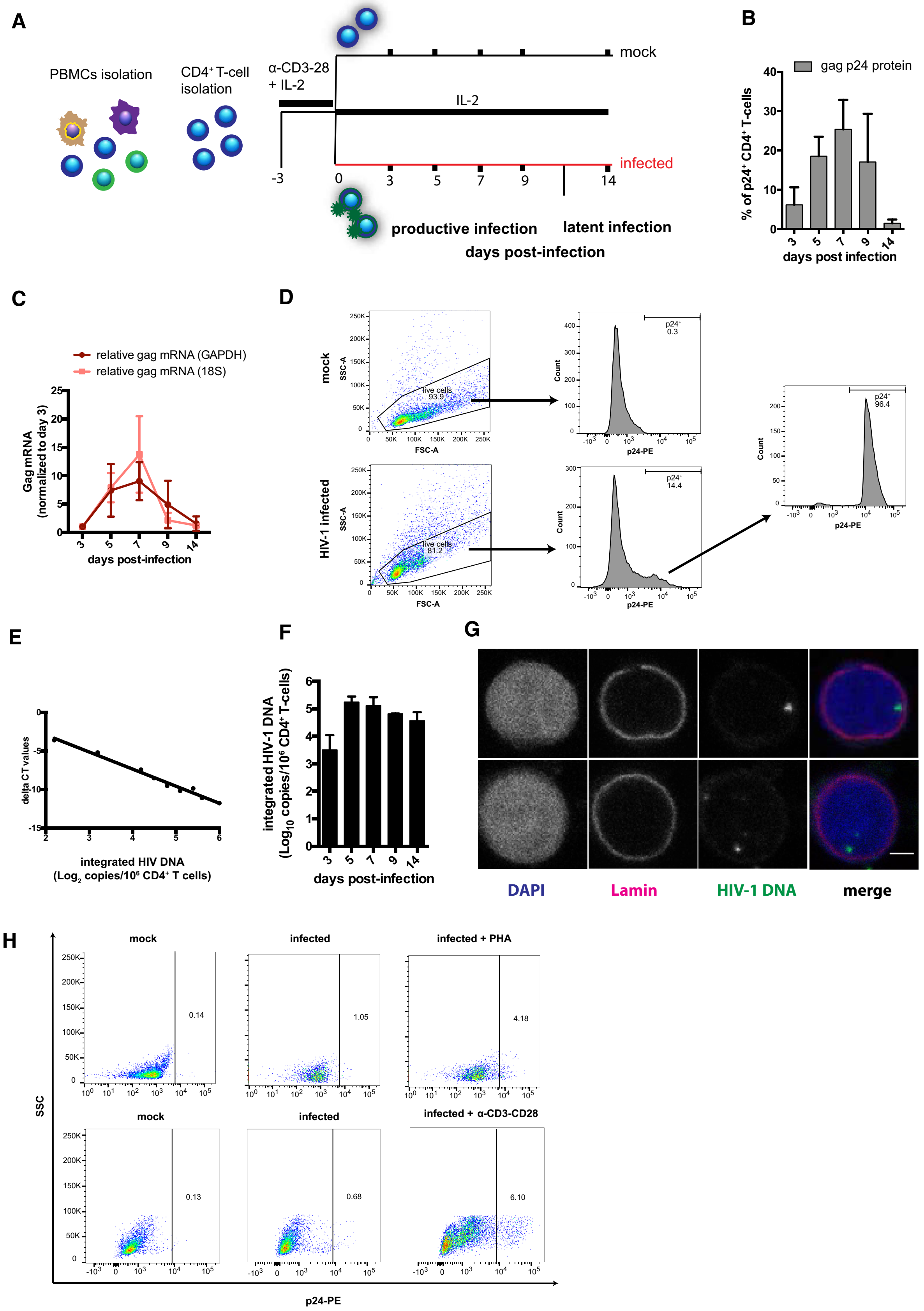

Figure EV1.

 © 2020 The Authors

Figure EV2. Influence of HIV-1 replication on antioxidant gene and protein expression.

A, B RNA-Seq (A) and proteomic (B) analyses of the relative expression over time of antioxidant genes and proteins in primary CD4+ T cells infected *in vitro* with HIV-1 or mock infected. (A) Heatmaps of the standardized expression of antioxidant genes in HIV-1-infected and mock-infected samples over time. Expression levels were standardized [(mean gene expression − SD)/SD] for each gene in each time point. Genes considered for further analysis in the paper are named on the right. (B) Boxplots of proteomics data illustrating the log_2 fold change expression for each time point in infected as compared to mock-infected cells. Median and 25–75 percentiles are depicted, while whiskers extend from the hinge to the highest or lowest value that is within 1.5 * IQR (inter-quartile range). Data beyond the end of the whiskers are outliers and plotted as points. Data were analyzed by Fisher test, for each time point, dots illustrate the pathway enrichment analysis of proteins up-regulated in infected vs. matched mock-infected controls. Dots size indicates the fraction of differentially expressed proteins in the pathway. All analyses were conducted using the pathway *GO cellular response to oxidative stress* (184 genes; GO:0034599). Number of donors = 3 biological replicates.

© 2020 The Authors

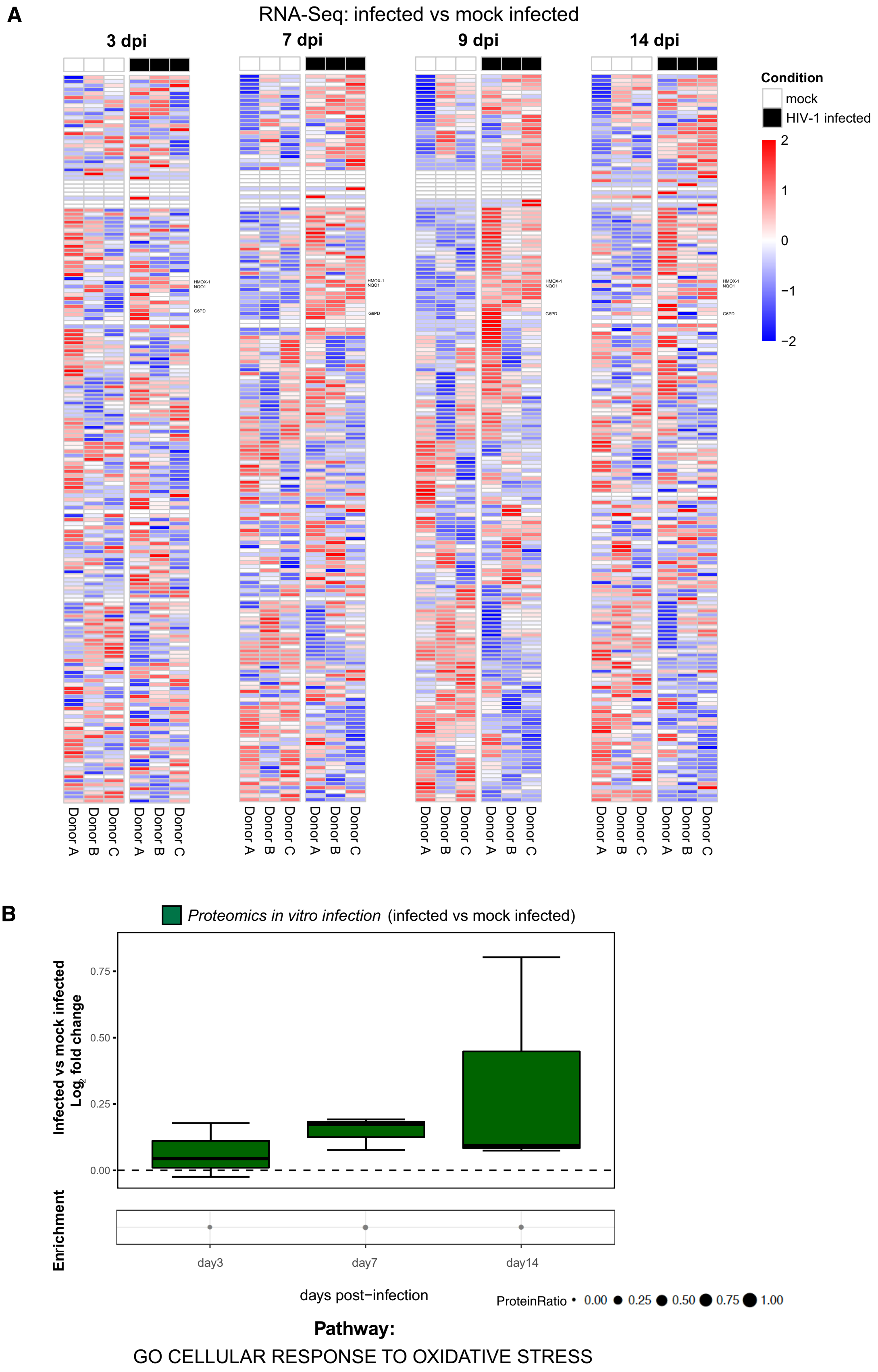

Figure EV2.

 © 2020 The Authors

Figure EV3. Redox stress markers and antioxidant responses upon HIV-1 infection in bulk and live primary CD4⁺ T cells.

A Time course of the protein expression of total and phosphorylated eIF2α in HIV-1-infected and mock-infected primary $CD4^+$ T cells.

B, C *Nrf2* (B) and *gag* (C) mRNA expression in HIV-1-infected Jurkat-TAg cells transfected with non-targeting siRNA (NC) or siRNAs targeting the *Nrf2* gene. Cells were infected 24 h post-transfection and assayed for mRNA expression by qPCR 48 h post-infection. Data (mean ± SEM; n = 4 technical replicates) were normalized using 18S as housekeeping gene and expressed as fold change in siRNA Nrf2-treated cells vs. NC control and analyzed by unpaired *t*-test.

D Relative (infected vs. mock-infected) mRNA levels of main targets of Nrf2 during the transition from productive (3–9 dpi) to latent (14 dpi) infection.

E Efficiency of magnetic enrichment of viable primary $CD4^+$ T cells. Live cells were isolated by negative selection (Annexin V binding), stained with a LIVE/DEAD dye before and after enrichment, and analyzed by flow cytometry.

F Relative (infected vs. mock-infected) mRNA levels of the targets analyzed in panel (D), performed on cells previously enriched for viability.

G Relative mRNA expression of Nrf2 downstream antioxidant targets in Jurkat T cells left untreated or treated for 24 h with 10 μM TPA.

Data information: Data in (F, G) were normalized using 18S, while data in (D) were normalized using GAPDH as housekeeping genes. After normalization, data were expressed $\log_2$ fold mRNA expression in infected vs. mock-infected (D, F) or untreated vs. TPA-treated cells (G) as in Livak and Schmittgen (2001). Data are shown as mean ± SEM of 3 biological replicates and were analyzed by two-way ANOVA followed by Tukey's post-test for multiple comparisons. $^*P < 0.05$; $^{**}P < 0.01$; $^{****}P < 0.0001$. Trx = thioredoxin; NQO1 = NAD(P)H [quinone] dehydrogenase 1; HMOX-1 = heme oxygenase 1; G6PD = glucose-6-phosphate dehydrogenase; GCLC = glutamate-cysteine ligase; TrxR1 = thioredoxin reductase 1.
Source data are available online for this figure.

© 2020 The Authors

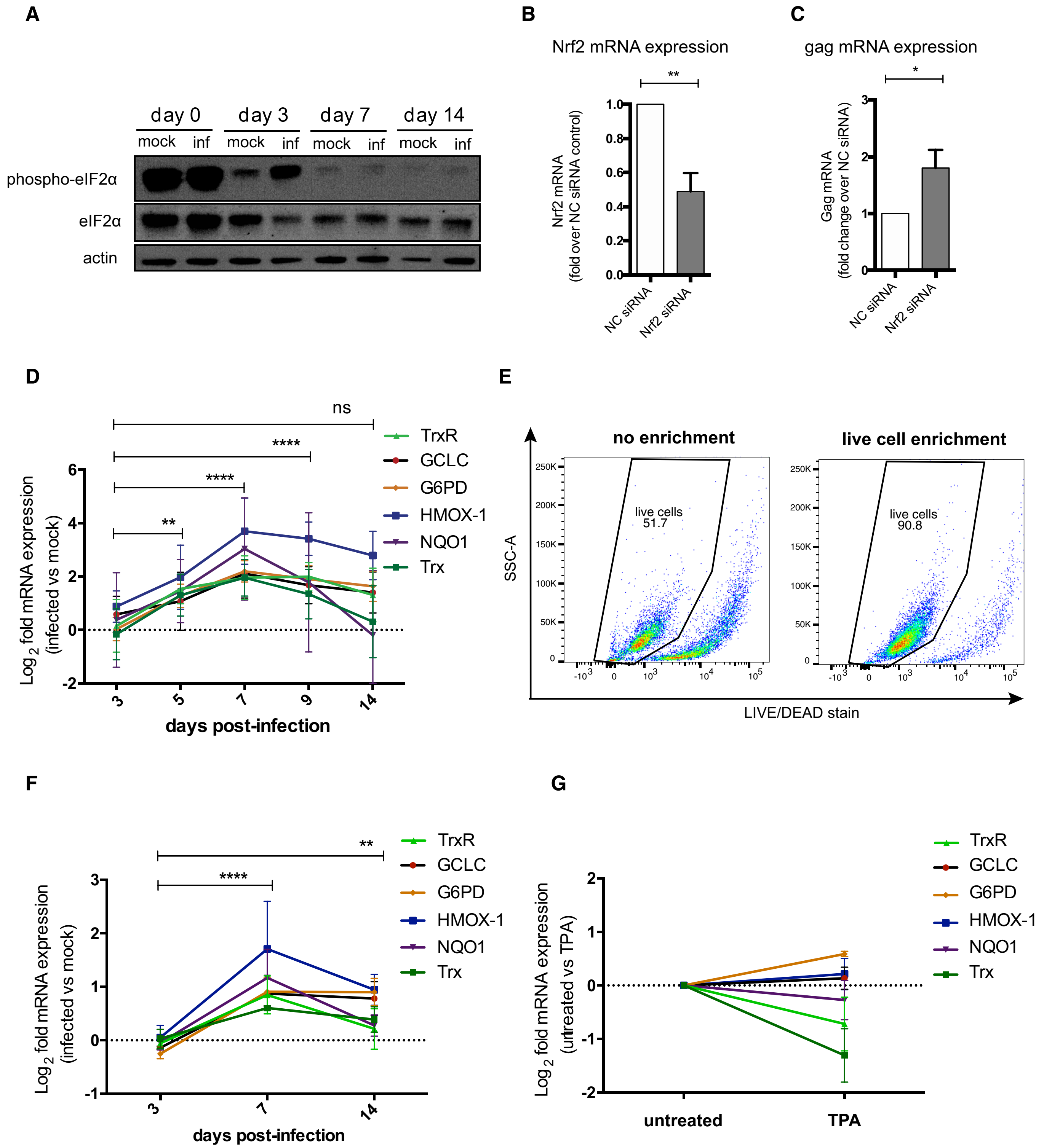

Figure EV3.

 © 2020 The Authors

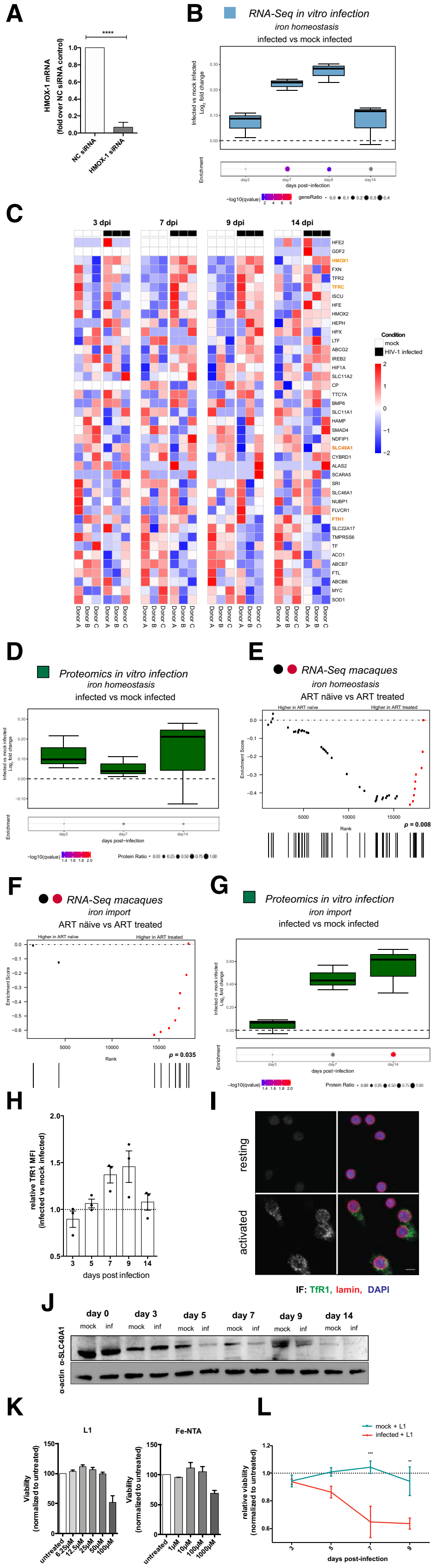

Figure EV4. Expression of iron homeostasis and import markers and effect of iron chelation on cell viability in HIV-1 infection.

A HMOX-1 mRNA expression in Jurkat-TAg cells transfected with non-targeting siRNA or siRNAs targeting the *HMOX-1* gene. Cells were collected 72 h post-transfection and analyzed by qPCR. Data (mean ± SEM; $n = 3$ technical replicates) were normalized using the NC control and analyzed by unpaired t-test. ****$P < 0.0001$.

B–G RNA-Seq (B, C, E, F) and proteomic (D, G) analysis of different time points of primary $CD4^+$ T cells infected *in vitro* with HIV-1 or mock infected (B, C, D, G) and PBMCs of macaques infected with SIVmac239 before and after suppression of viremia with ART (E, F). The gene sets used for the analyses are *GO cellular ion iron homeostasis* (46 genes, GO:0006879; B–E) and *GO iron ion import* (12 genes, GO:0097286; F, G). Data were analyzed by Fisher test (B, D, G; number of donors = 3 biological replicates) or GSEA (E, F, number of animals = 8). Boxplots in (B, D, G) illustrate the $\log_2$ fold change expression for each time point in infected as compared to mock-infected cells. Depicted in each boxplot are the median and 25–75 percentiles, while whiskers extend from the hinge to the highest or lowest value that is within 1.5 * IQR (inter-quartile range) of the hinge. Data beyond the end of the whiskers are outliers and plotted as points. Dots illustrate the pathway enrichment analysis of proteins up-regulated in infected cells. Dots are color-coded based on the enrichment q-values, and their size indicates the fraction of differentially expressed proteins in the pathways; gray dots are not statistically significant. Heatmaps in (C) depict the standardized expression of genes of the *GO cellular ion iron homeostasis pathway* in HIV-1-infected and mock-infected samples over time. Expression levels were standardized [(mean gene expression − SD)/SD] for each gene in each time point. Genes considered for further analysis in the paper are highlighted. Symbols in the GSEA enrichment plots (E, F) represent the position of the gene set members in the transcriptome ranked by differential expression between ART-naïve and ART-treated time points. Red color indicates leading edge genes.

H Relative TfR1 expression over time in HIV-1-infected vs. mock-infected $CD4^+$ T cells as measured by flow cytometry. Data are expressed as relative MFI (median fluorescence intensity) of TfR1 in HIV-1-infected vs. mock-infected cells (mean ± SEM; $n = 3$ biological replicates).

I Representative immunofluorescence image of TfR1 expression in $CD4^+$ T cells resting or activated for 72 h with α-CD3/CD28 beads. Scale bar = 10 μm.

J Protein expression over time of SLC40A1 in $CD4^+$ T cells infected with HIV-1 or mock infected as assessed by Western blot.

K Relative viability of $CD4^+$ T cells incubated for 48 h with various concentrations of the iron chelator deferiprone (L1) or the iron donor ferric nitriloacetate (Fe-NTA). Viability was measured using the MTT assay. Absorbance values were normalized over untreated controls and expressed as percentage (mean ± SEM, $n = 3$ biological replicates).

L Time course of the relative viability of HIV-1-infected or mock-infected $CD4^+$ T cells left untreated or treated with 50 μM L1. Viable cells were identified by flow cytometry through FSC/SSC gating (mean ± SEM, number of donors $n = 3$). **$P < 0.01$; ***$P < 0.001$.

Source data are available online for this figure.

© 2020 The Authors

Figure EV5. Oxidative stress and iron overload can alter PML stability.

A–C Nrf2 subcellular localization (A) and PML expression (B, C) in $CD4^+$ T cells left untreated or treated for 30 min with 100 μM H_2O_2. After washing away H_2O_2, cells were cultured for 24 h and harvested for biochemical fractionation (A) and Western blot (A, B), (WCE) whole cell extract; (CYT) cytoplasm; (NEsol) nuclear extract soluble; (NEtot) nuclear extract total. Data in (C) were quantified with Fiji-Image J (Schindelin *et al*, 2012), normalized to the housekeeping protein beta-actin, and expressed as fold change over untreated (mean ± SEM; n = 3 biological replicates). Data were analyzed by paired t-test. $^*P < 0.05$.

D–F PML (D) and PML NB (E, F) expression in $CD4^+$ T cells left untreated or treated for 48 h with 5 μM As_2O_3 or 500 μM $FeCl_3$ $6H_2O$ as measured by Western blot (D) or immunofluorescence (E, F). Box and whisker plots in (F) depict median and min to max range. n = number of cells. Scale bar 2 μm. Data were analyzed by Kruskal–Wallis test followed by Dunn's post-test. $^{***}P < 0.001$.

G Structural models of the coordination, in the PML-ring domain (pdb code: 5yuf), of the canonical binding atom (Zn^{2+}, blue sphere) compared to Fe(II) (orange sphere) and As3— (known to destabilize PML through direct binding (Zhang *et al*, 2010), purple sphere). Coordinating residues of zinc finger sites 1 (left panel) and 2 (right panel) are shown. Binding distances are indicated in Å (black for Fe(II) and Zn(II) and gray for As(III)). The predicted binding of Fe(III) overlaps with that of Fe(II), and it is excluded for simplicity.

Source data are available online for this figure.

© 2020 The Authors

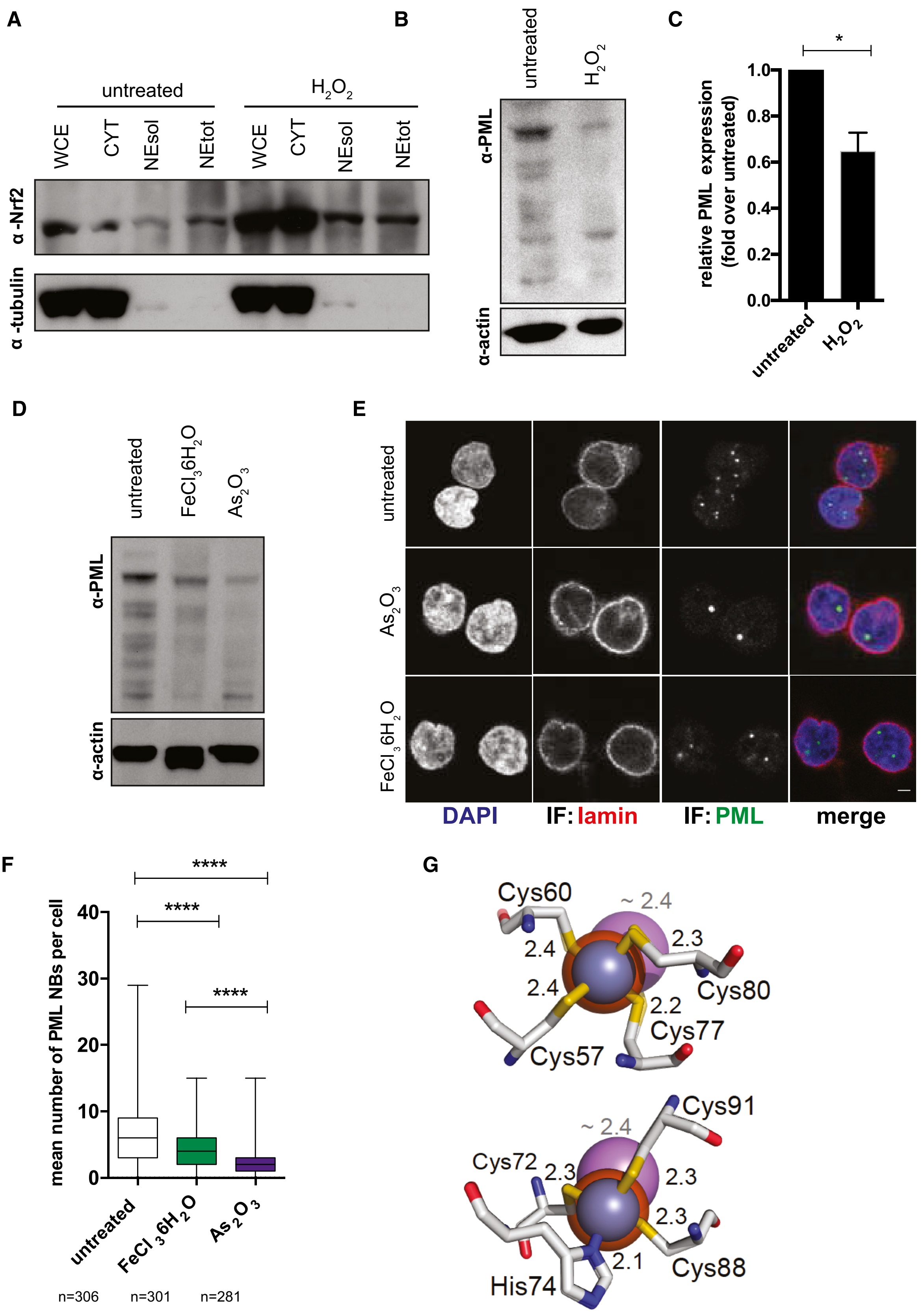
A
untreated
H2O2
WCE
CYT
NEsol
NEtot
α -Nrf2
α -tubulin
B
untreated
H2O2
α-PML
α-actin
C
*
relative PML expression (fold over untreated)
1.0
0.8
0.6
0.4
0.2
0.0
untreated
H2O2
D
untreated
FeCl3 6H2O
As2O3
α-PML
α-actin
E
untreated
As2O3
FeCl3 6H2O
DAPI
IF: lamin
IF: PML
merge
F
****
mean number of PML NBs per cell
40
30
20
10
0
untreated
FeCl3 6H2O
As2O3
n=306
n=301
n=281
G
Cys60
~ 2.4
2.3
2.4
2.4
Cys80
2.2
Cys77
Cys57
Cys91
~ 2.4
Cys72
2.3
2.3
2.3
2.1
Cys88
His74

Figure EV5.
