# Supplementary figures and images for: Alterations of redox and iron metabolism accompany the development of HIV latency
Source: EMBO J. 2020 Mar 11;39(9):e102209. doi: 10.15252/embj.2019102209 (PMC7196916; doi:10.15252/embj.2019102209)

Figure 2E

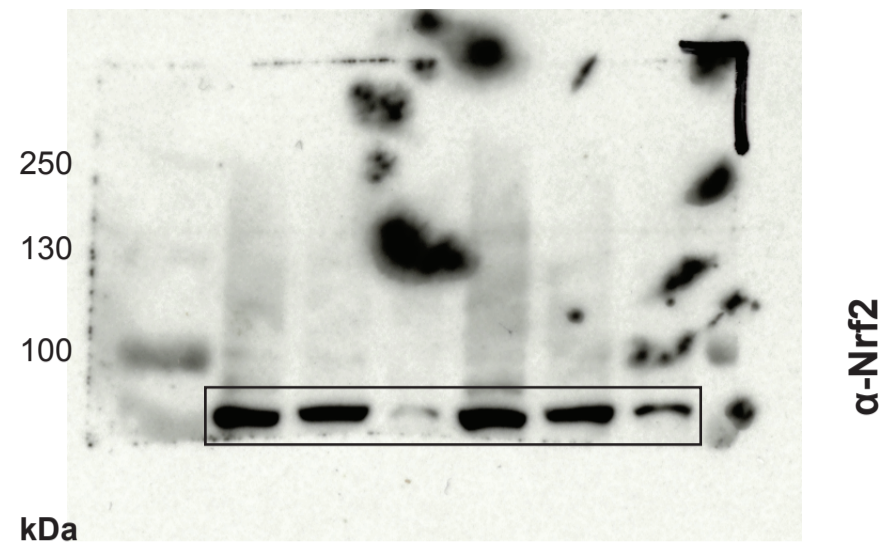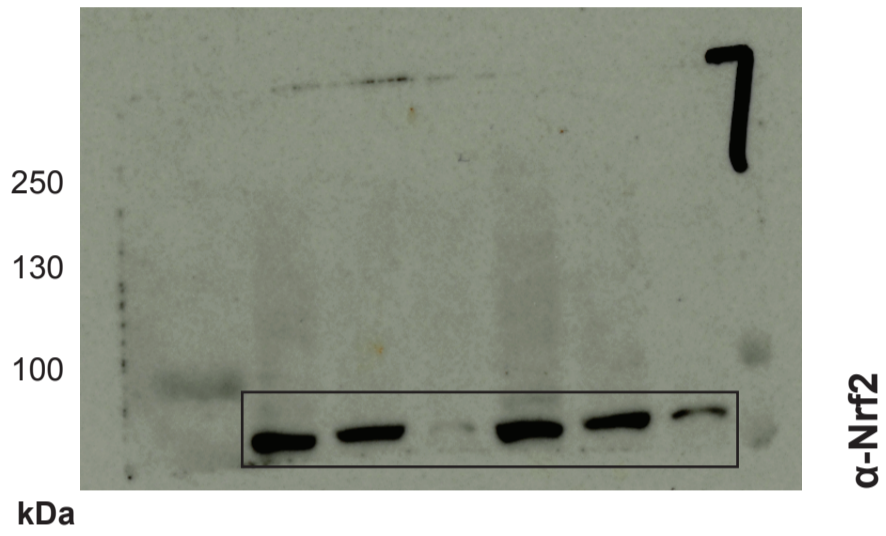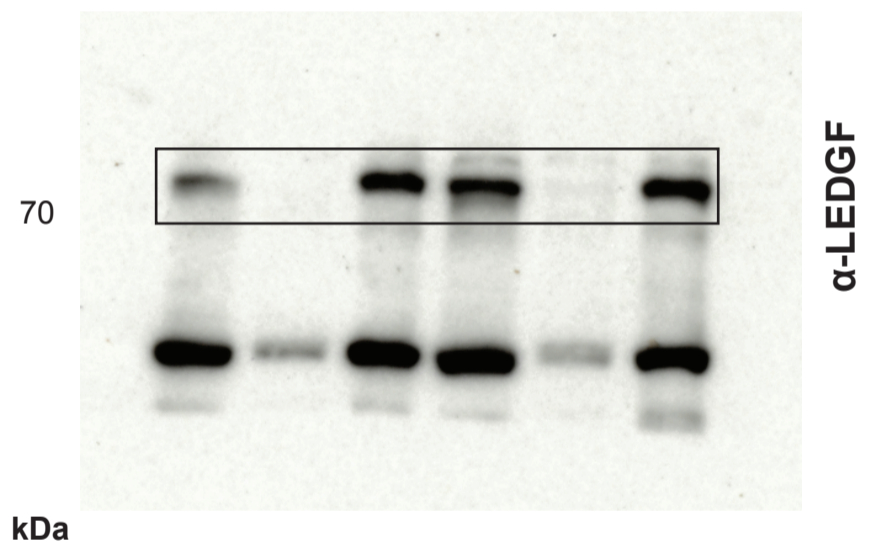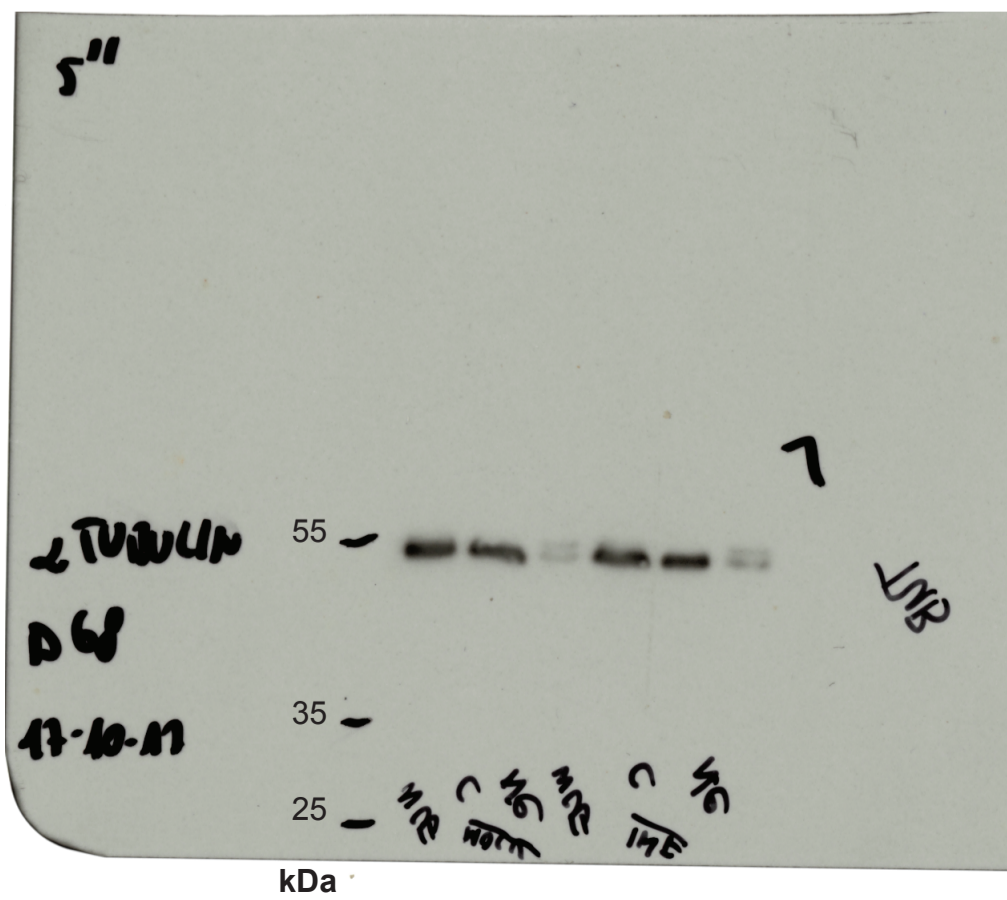

Figure 2H

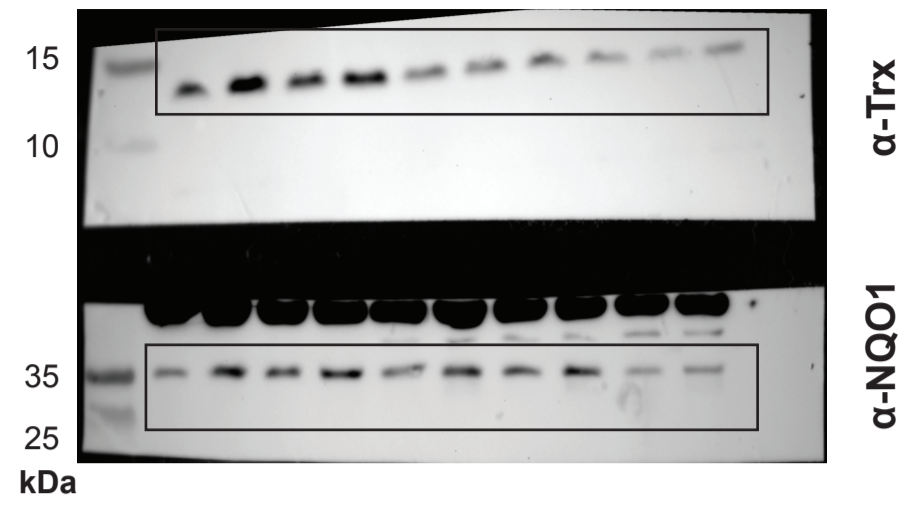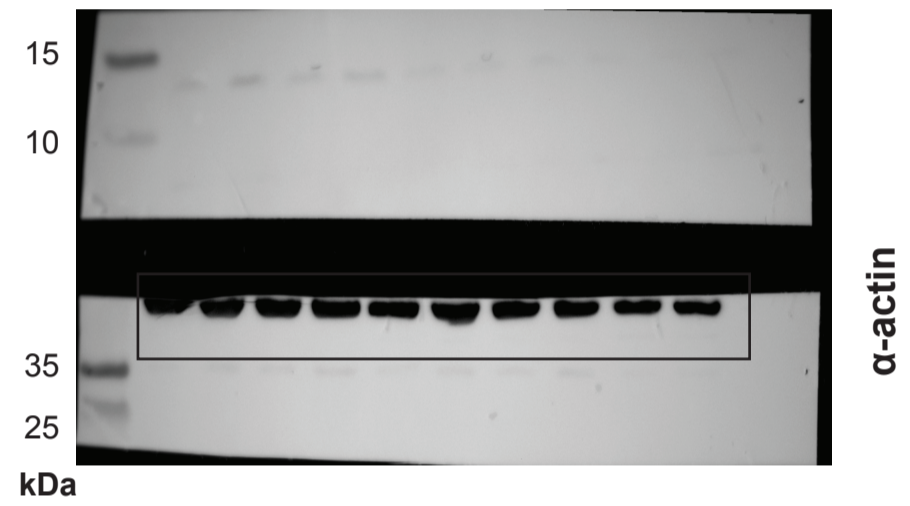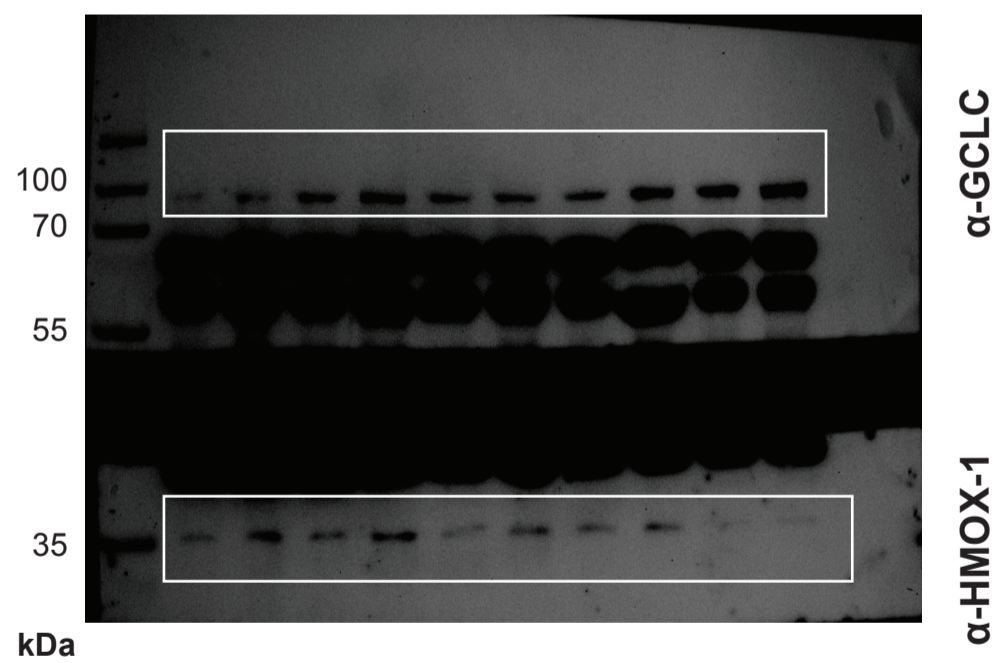

Supplement: Supplementary file 10 — Source Data for Figure 2 [file EMBJ-39-e102209-s008.zip › Fig_2E,_H.pdf]

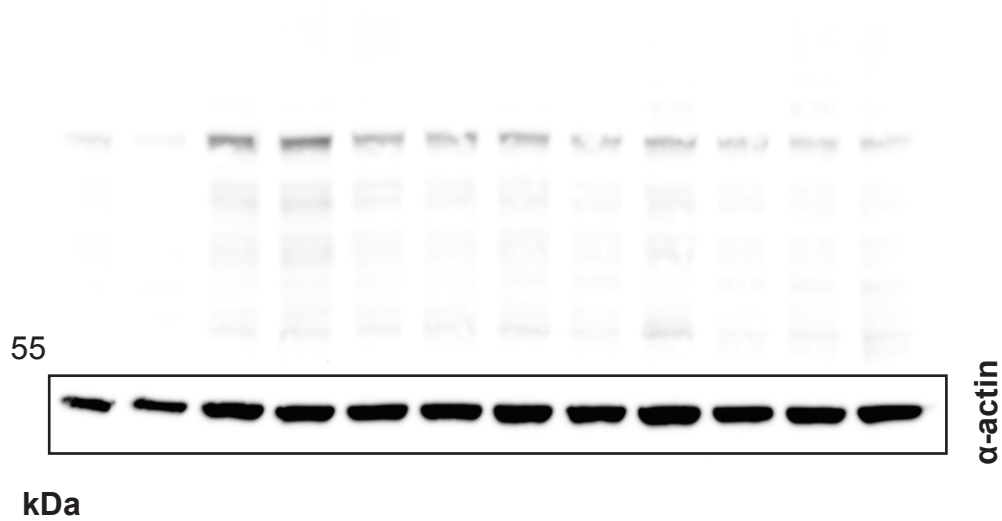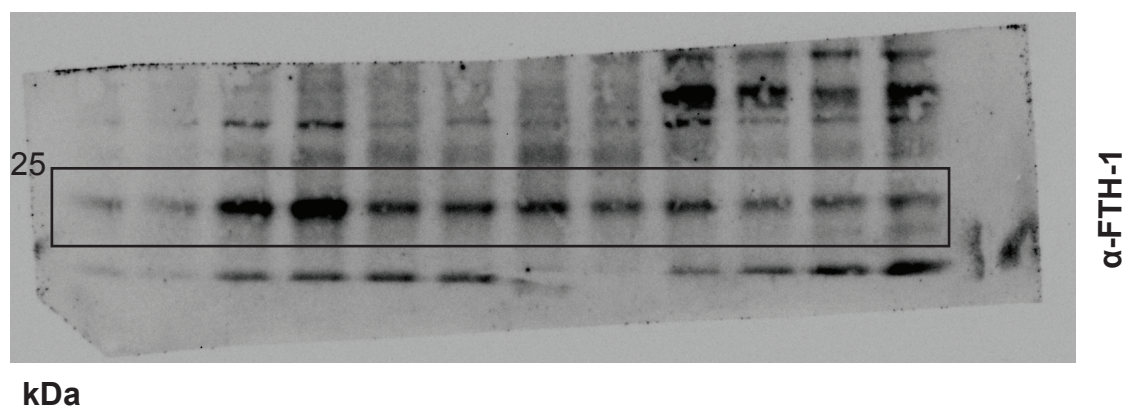

Supplement: Supplementary file 11 — Source Data for Figure 3 [file EMBJ-39-e102209-s009.zip › Fig_3.pdf]

**Figure 3E**

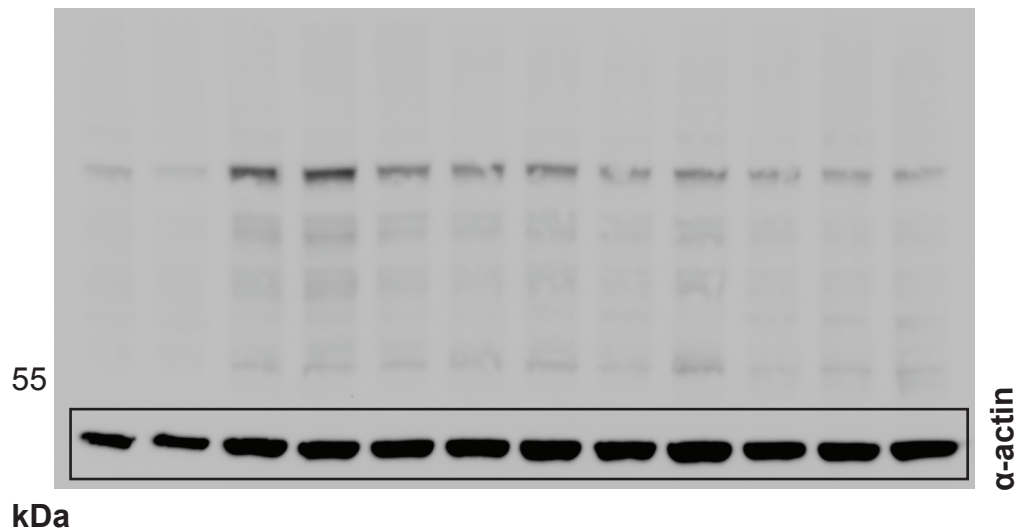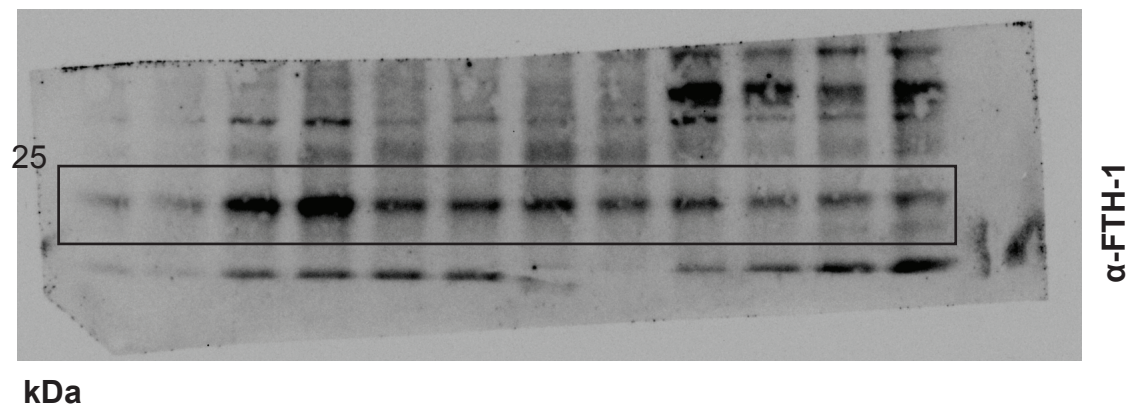

Supplement: Supplementary file 11 — Source Data for Figure 3 [file EMBJ-39-e102209-s009.zip › Fig_3E.pdf]

Figure 4C

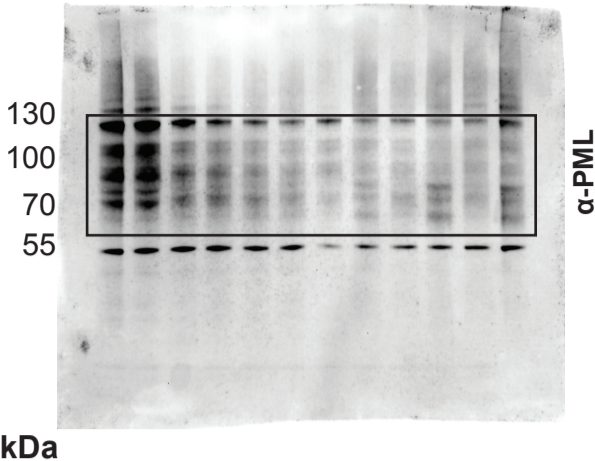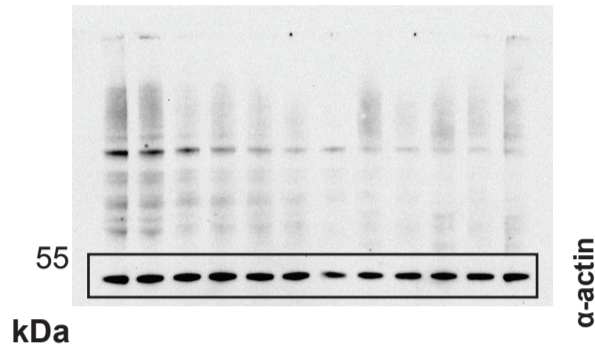

Figure 4E

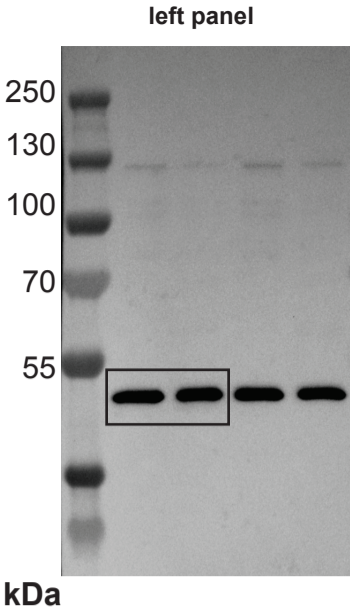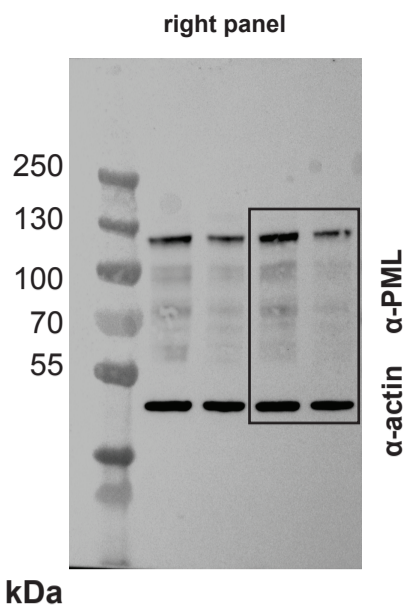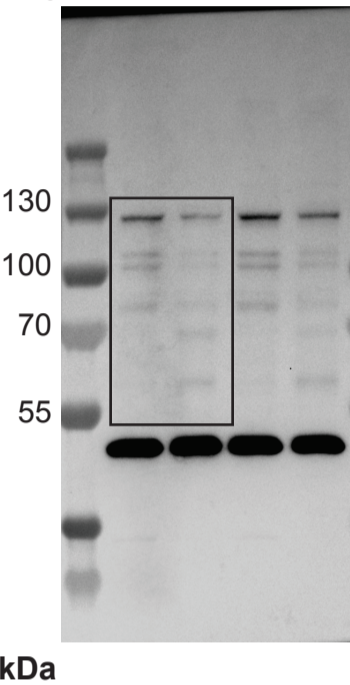

Figure 4F

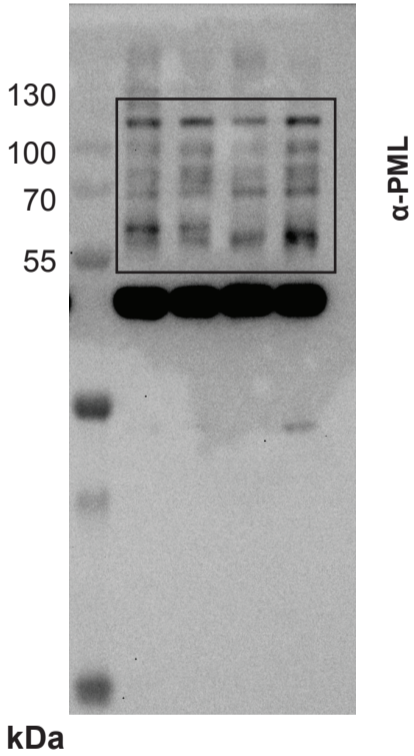

Figure 4G

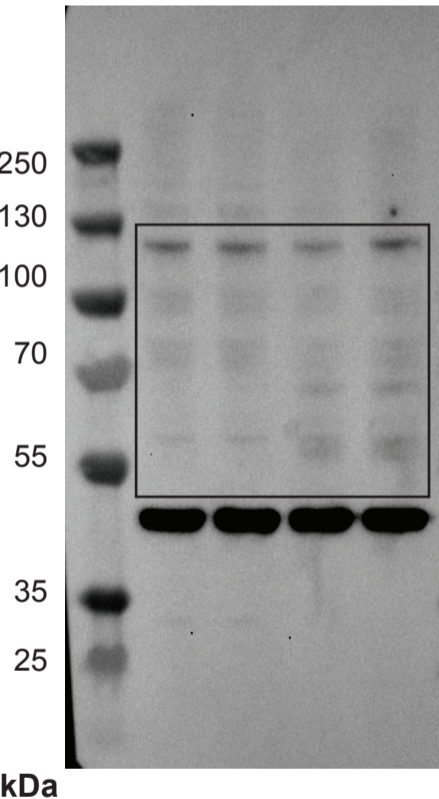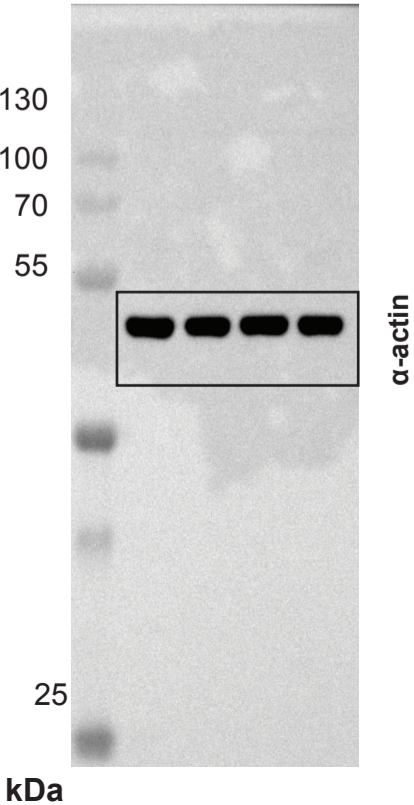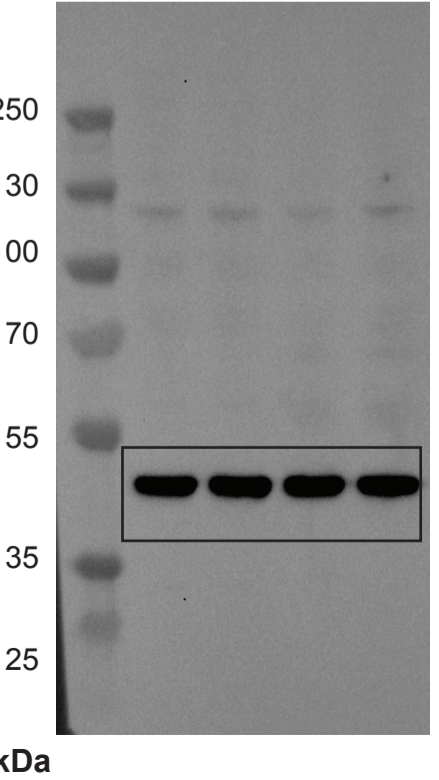

Supplement: Supplementary file 12 — Source Data for Figure 4 [file EMBJ-39-e102209-s010.pdf]
