# Supplementary material for: Alterations of redox and iron metabolism accompany the development of HIV latency
Source: EMBO J. 2020 Mar 11;39(9):e102209. doi: 10.15252/embj.2019102209 (PMC7196916; doi:10.15252/embj.2019102209)

Appendix Figure S1B

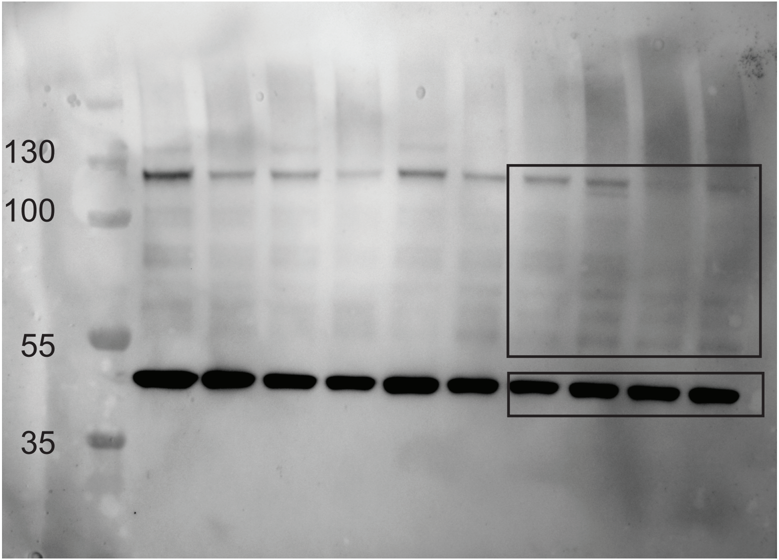

Supplement: Supplementary file 8 — Source Data for Expanded View and Appendix [file EMBJ-39-e102209-s011.zip › Appendix_Figure_Source_Data.zip!/Appendix Fig S1B.pdf]
